# Smoking and Quitting Behavior by Sexual Orientation: A Cross-Sectional Survey of Adults in England

**DOI:** 10.1093/ntr/ntaa042

**Published:** 2020-03-02

**Authors:** Sarah E Jackson, Jamie Brown, Igor Grabovac, Hazel Cheeseman, Ciaran Osborne, Lion Shahab

**Affiliations:** 1 Department of Behavioural Science and Health, University College London, London, UK; 2 Department of Clinical, Educational and Health Psychology, University College London, London, UK; 3 Department of Social and Preventive Medicine, Centre for Public Health, Medical University of Vienna, Vienna, Austria; 4 Action on Smoking and Health, London, UK

## Abstract

**Objective:**

To assess associations between sexual orientation and smoking and quitting behavior among adults in England.

**Methods:**

Data were collected from 112 537 adults (≥16 years) participating in a nationally representative monthly cross-sectional survey between July 2013 and February 2019. Sexual orientation was self-reported as heterosexual, bisexual, lesbian/gay, or prefer-not-to-say. Main outcomes were smoking status, e-cigarette use, cigarettes per day, time to first cigarette, motivation to stop smoking, motives for quitting, use of cessation support, and past‐year quit attempts. Associations were analyzed separately for men and women using multivariable regression models adjusted for relevant covariates.

**Results:**

Smoking prevalence is now similar between gay (21.6%), prefer-not-to-say (20.5%) and heterosexual men (20.0%), and lesbian (18.3%) and heterosexual women (16.9%), but remains higher among bisexual men (28.2%, adjusted odds ratio [OR_adj_] = 1.41, 95% confidence interval [CI] = 1.11 to 1.79) and bisexual women (29.8%, OR_adj_ = 1.64, 95% CI = 1.33 to 2.03) and lower among prefer-not-to-say women (14.5%, OR_adj_ = 0.85, 95% CI = 0.72 to 0.99). Among smokers, bisexuals were less addicted than heterosexuals, with bisexual men smoking fewer cigarettes per day (*B*_adj_ = −2.41, 95% CI = −4.06 to −0.75) and bisexual women less likely to start smoking within 30 min of waking (OR_adj_ = 0.66, 95% CI = 0.45 to 0.95) than heterosexuals. However, motivation to stop smoking and quit attempts did not differ significantly.

**Conclusions:**

In England, differences in smoking prevalence among people with different sexual orientations have narrowed, primarily driven by a larger decline in smoking rates among sexual minority groups than heterosexuals. Bisexual men and women remain more likely to smoke but have lower levels of addiction while being no less likely to try to quit.

**Implications:**

This population-based study provides an up-to-date picture of smoking and quitting behavior in relation to sexual orientation among adults in England. Findings suggest that widely documented disparities in smoking prevalence have narrowed over recent years, with gay men and lesbian women no longer significantly more likely to smoke than heterosexuals, although smoking remains more common among bisexual men and women. Insights into differences in level of addiction, use of cessation support, and motives for quitting may help inform the development of targeted interventions to further reduce smoking among sexual minority groups.

## Introduction

Despite the substantial progress that has been achieved in reducing smoking prevalence over recent decades, tobacco use remains one of the leading causes of premature death and disability worldwide.^1^ With disproportionately high rates of smoking in certain population groups, it is a key contributor to health inequalities.^2^ In England, National Institute of Health and Care Excellence (NICE) guidelines published in 2018 emphasize the need for high-prevalence groups to be targeted and prioritized in smoking cessation initiatives and services.^3^ One group identified by NICE as a priority is sexual minorities (including lesbian, gay, and bisexual [LGB] people).^3^ A better understanding of smoking rates, motivation to quit, and difficulty quitting in this population group is required for the development of targeted interventions.

Evidence on the LGB population has traditionally been limited by a lack of routine monitoring of sexual orientation in public services and epidemiological research.^4^ As such, there is relatively little robust data on smoking behavior in this population group, particularly outside of the United States. Most studies that have examined the association between sexual orientation and smoking status have relied on small convenience samples,^5^ although there have recently been several larger, representative studies conducted in the United States^6–10^ and Australia.^11^ The majority have observed higher rates of smoking among sexual minority groups,^5–15^ although a large, representative study of adults in England found no significant difference after adjustment for other sociodemographic variables.^16^

There are several factors that may contribute to higher smoking prevalence among sexual minorities. Smoking is a socially contagious behavior and is initiated and maintained through social networks.^17^ For many LGB people, safe places for social gathering have traditionally been bars and similar establishments where there is a culture of smoking.^18^ The tobacco industry has also been known to specifically target sexual minority groups.^19^ For some LGB adults, smoking may be a mechanism for coping with minority stress.^20,21^ Given the high levels of social exclusion experienced by sexual minority groups, it is also plausible that smoking persists due to fear of exclusion from the social group if the behavior stops.^22,23^

The extant literature on tobacco use in sexual minorities has predominantly focused on smoking status, with little exploration of different aspects of smoking behavior that may be relevant to the design of targeted services and interventions (eg, level of addiction, motivation to quit, or success in quitting). To our knowledge, a study we conducted on data collected up to May 2016 represents the only representative study in England to report on differences in smoking characteristics between LGB and heterosexual smokers.^16^ Results indicated no notable differences in male smokers; among women, bisexuals appeared to be less dependent than heterosexuals but there was no significant difference in motivation to quit or the prevalence of past-year quit attempts.^16^ There is a need to update these figures and, given the slow accumulation of data on this minority population, a first opportunity to examine several variables relevant to the design of targeted interventions for which there was previously insufficient power (eg, motives for quitting, use of e-cigarettes). US studies that have explored differences in e-cigarette use in relation to sexual orientation have observed higher rates of ever and current e-cigarette use among sexual minorities.^6,8,10^

This study was therefore designed to update and extend the evidence base by providing a detailed assessment of associations between sexual orientation and smoking, use of e-cigarettes, and quitting behavior. Data were drawn from a large, representative sample of the adult population in England, with data collected monthly between 2013 and 2019.

Specifically, we aimed to address the following research questions:

1. How does the prevalence of smoking in adults who identify as lesbian, gay, bisexual, and prefer-not-to-say compare with those who identify as heterosexual, adjusting for a range of sociodemographic factors?
2. To what extent has smoking prevalence changed over time in adults who identify as lesbian, gay, bisexual, and prefer-not-to-say in comparison with those who identify as heterosexual?
3. Overall, and by smoking status, how does the prevalence of e-cigarette use in adults who identify as lesbian, gay, bisexual, and prefer-not-to-say compare with those who identify as heterosexual, adjusting for a range of sociodemographic factors?
4. Among current smokers, how does the prevalence of high motivation to quit smoking and markers of cigarette addiction in adults who identify as lesbian, gay, bisexual, and prefer-not-to-say compare with those who identify as heterosexual, adjusting for a range of sociodemographic factors?
5. Among past‐year smokers, how does the prevalence of a quit attempt in the past year in adults who identify as lesbian, gay, bisexual, and prefer-not-to-say compare with those who identify as heterosexual, adjusting for a range of sociodemographic factors?
6. Among past‐year smokers who have made at least one quit attempt in the past year, how do the motives for quitting, use of smoking cessation aids and success rates of adults who identify as lesbian, gay, bisexual, and prefer-not-to-say compare with those who identify as heterosexual, adjusting for a range of sociodemographic factors?

## Method

### Design

The Smoking Toolkit Study (STS) is an ongoing monthly cross-sectional survey of representative samples of adults (≥16 years) in England. It is designed to provide insights into population-wide influences on smoking and cessation by monitoring trends on a range of variables relating to smoking.^24^ It uses a form of random location sampling to select a new sample of approximately 1700 adults aged ≥16 years each month. Participants complete a face‐to‐face computer‐assisted survey with a trained interviewer. Comparisons with national data indicate that key sociodemographic variables and smoking prevalence are nationally representative.^24^ Ethical approval for the Smoking Toolkit Study was granted originally by the UCL Ethics Committee (ID 0498/001), and participants provided full informed consent. The data are not collected by UCL and are anonymized when received by UCL.

### Population

The present study used aggregated data from respondents to the STS survey between July 2013 (the first wave to ask about sexual orientation) and February 2019 (the most recent wave of data available at the time of analysis).

### Measures

#### Explanatory

Sexual orientation was self-reported as (1) bisexual; (2) gay man/homosexual; (3) gay woman/lesbian; (4) heterosexual/straight; or (5) prefer-not-to-say. This measure has been validated by the government Office for National Statistics in England.^25^

#### Outcomes

We examined the following outcomes: (1) in all adults: the prevalence of cigarette smoking and the prevalence of e-cigarette use (overall and in relation to smoking status: current smoker, recent ex-smoker [<1 year], long-term ex-smoker [≥1 year], never-smoker); (2) in current smokers: mean number of cigarettes smoked per day (CPD) and the proportion who smoke within 30 min of waking (two markers of cigarette addiction), and high motivation to stop (“really want and plan to stop within 3 months”)^26^; (3) in past‐year smokers: the proportion who made a serious attempt to quit in the past year; and (4) in smokers who made a quit attempt in the past year: motives for quitting, the proportion who used cessation support (behavioral, nicotine replacement therapy (NRT) over the counter (OTC), electronic cigarettes (e‐cigarettes) or prescription medication) and quit success (ie, the proportion not currently smoking).

#### Potential Confounders

Potential confounders included gender, age, ethnicity (based on skin color and national background, collapsed to white/nonwhite), social grade (an occupational index of socioeconomic position, categorized as ABC1, which includes managerial, administrative and professional and occupations, vs. C2DE, which includes semi‐routine and routine occupations, manual occupations, never workers, and long‐term unemployed^27^), marital status (married, civil partnership, or living with partner: yes/no), disability (yes/no), and survey year.

### Statistical Analysis

The analysis plan was preregistered on Open Science Framework (https://osf.io/25nkq/).

Data were weighted using rim (marginal) weighting to match the English population profile relevant to the time each monthly survey was conducted on dimensions of age, social grade, region, tenure, ethnicity, and working status within sex.

Descriptive data on all outcomes and potential confounders are provided for each of the sexual orientation categories. We used descriptive statistics to summarize annual trends in smoking prevalence between 2013 and 2019 in relation to sexual orientation. We used linear regression for continuous outcomes and logistic regression for binary outcomes to analyze associations between sexual orientation and our outcomes of interest, with and without adjustment for potential confounders. The reference category was heterosexual/straight. Results are reported as unstandardized *B* coefficients or odds ratios (OR) with 95% confidence intervals (CIs). On the basis of the previous study conducted in this sample, which indicated systematic differences in the relationship between sexual orientation and smoking by gender,^16^ all results are reported separately for men and women with the exception of smoking prevalence trends which are reported for both sexes combined to maximize sample numbers at each time point. Missing data were removed on a per-analysis basis for each outcome.

Where differences on key outcomes (smoking prevalence, motivation to stop smoking, and quit attempts) between LGB and heterosexual groups were not statistically significant, Bayes factors (BF) were calculated to determine whether results are supportive of the null hypothesis (ie, no difference between groups), the alternative hypothesis (ie, a difference between groups), or are insensitive to detect a difference. The use of BFs in the interpretation of nonsignificant findings is gaining momentum in addiction science, with leading journals and researchers in the field advocating their use as a supplement to frequentist statistics in order to more accurately characterize the evidence for competing hypotheses.^28–30^ We used a conservative approach with alternative hypotheses represented by a half-normal distribution. The half-normal distribution considers values close to the null most plausible, which can make it hard to distinguish the alternative hypothesis from the null; thus, any BF that does clearly distinguish between the hypotheses provides good evidence to support our conclusion of no difference.^31^ The absolute expected effect size for categorical outcomes was set to OR = 1.5 in the observed direction (ie, OR = 1.5 for observed ORs >1 and OR = 0.67 for observed ORs <1) and for continuous outcomes set to beta = 0.5 (ie, beta = 0.5 for observed betas >0 and beta = −0.5 for observed betas <0). This expected effect size was based on previous studies that have examined smoking behavior in relation to sexual orientation.^16^ BFs ≥3 can be interpreted as evidence for the alternative hypothesis (and against the null), BFs ≤1/3 as evidence for the null hypothesis, and BFs between 1/3 and 3 suggest the data are insensitive to distinguish the alternative hypothesis from the null.^31,32^

All analyses were conducted in SPSS v.24, with the exception of the BFs which were calculated using an online calculator (http://www.lifesci.sussex.ac.uk/home/Zoltan_Dienes/inference/Bayes.htm).

## Results

### Sample Characteristics

Our sample included 112 537 adults (≥16 years) who participated in the STS between July 2013 and February 2019. The majority (91.5%, *n* = 102 999) identified as heterosexual, 1.1% (*n* = 1216) identified as bisexual, 2.4% (*n* = 2666) identified as lesbian/gay, and 5.0% (*n* = 5657) preferred not to disclose their sexual orientation. Sample characteristics are summarized in Table 1. Compared with those who identified as heterosexual, participants who identified as lesbian/gay or bisexual were more likely to be younger and less likely to be married, in a civil partnership, or living with someone. Those who identified as lesbian/gay were more likely to be white, and those who identified as bisexual were more likely be from social grades C2DE and to report a disability.

**Table 1.** Sample Characteristics, Overall and by Sexual Orientation

	Whole sample	Heterosexual	Bisexual	Lesbian/gay	Prefer-not-to-say
All adults (*n*)	112 538	102 998	1217	2667	5656
Female	50.9 (57 330)	50.9 (52 457)	56.3 (685)	47.9 (1277)	51.5 (2911)
Age (years)					
16–24	13.8 (15 478)	13.5 (13 893)	35.2 (428)	16.7 (446)	12.6 (711)
25–34	16.9 (19 023)	16.8 (17 256)	23.9 (291)	19.7 (525)	16.8 (951)
35–44	16.5 (18 552)	16.6 (17 113)	16.2 (197)	15.2 (406)	14.8 (836)
45–54	17.4 (19 610)	17.6 (18 114)	9.3 (113)	16.8 (449)	16.5 (934)
55–64	14.1 (15 878)	14.2 (14 618)	6.8 (83)	12.3 (327)	15.0 (850)
65+	21.3 (23 997)	21.4 (22 004)	8.6 (105)	19.3 (514)	24.3 (1374)
White ethnicity	86.4 (96 761)	86.6 (88 956)	83.1 (1007)	90.7 (2417)	79.8 (4381)
Social grade C2DE	45.1 (50 748)	45.0 (46 300)	48.0 (583)	45.3 (1209)	47.0 (2656)
Married/cohabiting	57.9 (36 245)	58.6 (33 154)	43.2 (381)	53.3 (1137)	51.1 (1573)
Disability	10.9 (12 183)	10.8 (11 029)	16.5 (1198)	11.8 (2632)	12.1 (5334)
Men (*n*)	55 166	50 522	525	1385	2734
Age (years)					
16–24	14.5 (8010)	14.3 (7232)	26.9 (141)	18.8 (261)	13.8 (376)
25–34	17.5 (9639)	17.2 (8702)	25.3 (133)	20.6 (285)	19.0 (519)
35–44	16.6 (9183)	16.7 (8449)	15.4 (81)	15.4 (213)	16.1 (440)
45–54	17.6 (9719)	17.8 (8985)	12.4 (65)	16.7 (231)	16.0 (438)
55–64	14.1 (7790)	14.2 (7163)	9.0 (47)	12.0 (166)	15.1 (414)
65+	19.6 (10 825)	19.8 (9991)	11.0 (58)	16.5 (229)	20.0 (547)
White ethnicity	84.8 (46 555)	85.1 (42 832)	78.6 (411)	90.7 (1257)	77.3 (2055)
Social grade C2DE	45.1 (24 861)	45.0 (22 718)	48.0 (251)	40.9 (567)	48.5 (1325)
Married/cohabiting	61.0 (18 717)	61.8 (17 160)	48.0 (180)	52.0 (545)	56.3 (832)
Disability	10.0 (5466)	9.8 (4929)	16.9 (87)	11.3 (155)	11.5 (295)
Women (*n*)	57 330	52 457	686	1277	2910
Age (years)					
16–24	13.0 (7463)	12.7 (6659)	41.4 (284)	14.5 (185)	11.5 (335)
25–34	16.3 (9373)	16.3 (8552)	22.6 (155)	18.6 (238)	14.7 (428)
35–44	16.3 (9365)	16.5 (8663)	16.9 (116)	15.0 (191)	13.6 (395)
45–54	17.2 (9881)	17.4 (9123)	6.9 (47)	17.1 (218)	16.9 (493)
55–64	14.1 (8081)	14.2 (7450)	5.2 (36)	12.5 (160)	14.9 (435)
65+	23.0 (13 167)	22.9 (12 010)	7.0 (48)	22.3 (285)	28.3 (824)
White ethnicity	87.9 (50 176)	88.1 (46 109)	86.7 (592)	90.8 (1157)	82.2 (2318)
Social grade C2DE	45.1 (25 868)	44.9 (23 572)	48.0 (329)	50.1 (640)	45.6 (1327)
Married/cohabiting	54.8 (17 504)	55.6 (15 984)	40.3 (201)	54.4 (588)	46.1 (731)
Disability	11.8 (6712)	11.7 (6099)	16.0 (108)	12.3 (156)	12.7 (349)

Data are presented as % (*n*). Weighted data shown. Numbers may not sum to the total sample number due to missing data; valid percentages are given for ease of interpretation.

### Associations With Smoking and Quitting Behavior

Associations between sexual orientation and smoking and cessation outcomes are summarized in Table 2 (men) and Table 3 (women).

**Table 2.** Smoking and Cessation Behavior in Relation to Sexual Orientation in Men

	Heterosexual	Bisexual	Gay	Prefer-not-to-say
All adults (*n*)	50 522	525	1385	2734
Current smoking				
%	20.0	28.2	21.6	20.5
OR [95% CI] * p*	1 (ref)	1.58 [1.30 to 1.91] <.001	1.10 [0.97 to 1.25] .152	1.03 [0.94 to 1.13] .540
OR_adj_ [95% CI] * p*	1 (ref)	1.41 [1.11 to 1.79] .005	0.98 [0.83 to 1.15] .762	0.92 [0.80 to 1.07] .282
E-cigarette use				
%	5.9	8.8	7.1	6.0
OR [95% CI] * p*	1 (ref)	1.52 [1.12 to 2.07] .007	1.21 [0.98 to 1.49] .076	1.02 [0.87 to 1.20] .812
OR_adj_ [95% CI] * p*	1 (ref)	1.29 [0.89 to 1.87] .184	0.93 [0.71 to 1.21] .576	0.99 [0.78 to 1.25] .921
Current smokers (*n*)	10 068	148	296	556
Cigarettes per day				
Mean (SD)	11.8 (8.6)	10.7 (12.1)	10.5 (8.2)	11.1 (8.0)
*B* [95% CI] * p*	Ref	−1.06 [−2.50 to 0.38] .148	−1.24 [−2.28 to −0.21] .019	−0.71 [−1.48 to 0.06] .071
*B*_adj_ [95% CI] * p*	Ref	−2.41 [−4.06 to −0.75] .004	−1.26 [−2.46 to −0.06] .040	−0.60 [−1.70 to 0.50] .281
First smoke within 30 min of waking				
%	46.6	41.9	45.9	47.4
OR [95% CI] * p*	1 (ref)	0.83 [0.60 to 1.16] .273	0.97 [0.77 to 1.23] .826	1.03 [0.87 to 1.22] .736
OR_adj_ [95% CI] * p*	1 (ref)	0.75 [0.49 to 1.14] .173	0.97 [0.73 to 1.31] .862	1.28 [0.98 to 1.67] .070
High motivation to stop				
%	14.2	12.8	14.5	12.8
OR [95% CI] * p*	1 (ref)	0.90 [0.55 to 1.45] .659	1.04 [0.75 to 1.44] .831	0.89 [0.69 to 1.15] .359
OR_adj_ [95% CI] * p*	1 (ref)	0.65 [0.33 to 1.28] .212	0.98 [0.65 to 1.49] .927	0.94 [0.64 to 1.39] .760
E-cigarette use				
%	19.1	22.3	18.8	20.0
OR [95% CI] * p*	1 (ref)	1.23 [0.83; 1.81] .303	0.98 [0.73; 1.32] .910	1.07 [0.86; 1.32] 0.550
OR_adj_ [95% CI] * p*	1 (ref)	1.22 [0.77 to 1.95] .403	0.80 [0.54 to 1.18] .258	1.13 [0.82 to 1.56] .456
Former smokers (*n*)	10 220	73	201	499
E-cigarette use				
%	9.0	13.7	18.4	8.6
OR [95% CI] * p*	1 (ref)	1.59 [0.81 to 3.12] .182	2.30 [1.61 to 3.30] <.001	0.95 [0.69 to 1.30] .734
OR_adj_ [95% CI] * p*	1 (ref)	0.84 [0.33 to 2.19] .727	2.05 [1.29 to 3.25] .002	0.92 [0.59 to 1.44] .717
Never-smokers (*n*)	30 178	304	884	1672
E-cigarette use				
%	0.5	1.0	0.5	0.6
OR [95% CI] * p*	1 (ref)	1.93 [0.58 to 6.40] .281	1.01 [0.38 to 2.66] .992	1.22 [0.64 to 2.36] .544
OR_adj_ [95% CI] * p*	1 (ref)	1.87 [0.56 to 6.28] .311	0.70 [0.23 to 2.16] .537	1.09 [0.47 to 2.54] .849
Past-year smokers (*n*)	10 536	165	312	562
Past-year quit attempt				
%	32.0	38.2	37.8	32.6
OR [95% CI] * p*	1 (ref)	1.32 [0.96 to 1.82] .084	1.30 [1.03 to 1.64] .026	1.03 [0.86 to 1.23] .759
OR_adj_ [95% CI] * p*	1 (ref)	1.19 [0.80 to 1.76] .402	1.26 [0.94 to 1.70] .124	1.30 [0.99 to 1.71] .062
Past-year smokers who made a quit attempt (*n*)	3368	64	119	183
Motivated by GP advice				
%	15.2	28.6	18.5	19.1
OR [95% CI] * p*	1 (ref)	2.21 [1.27 to 3.86] .005	1.24 [0.77 to 2.00] .372	1.32 [0.90 to 1.93] .159
OR_adj_ [95% CI] * p*	1 (ref)	5.21 [2.40 to 11.30] <.001	2.65 [1.44 to 4.86] .002	2.08 [1.16 to 3.73] .013
Motivated by health concerns				
%	49.6	35.9	48.3	44.3
OR [95% CI] * p*	1 (ref)	0.57 [0.34 to 0.95] .033	0.94 [0.65 to 1.36] .752	0.81 [0.60 to 1.10] .172
OR_adj_ [95% CI] * p*	1 (ref)	0.51 [0.27 to 0.98] .042	0.59 [0.37 to 0.96] .032	0.64 [0.41 to 1.00] .050
Motivated by cost				
%	19.4	18.8	26.1	15.8
OR [95% CI] * p*	1 (ref)	0.93 [0.49 to 1.77] .823	1.46 [0.96 to 2.22] .077	0.79 [0.53 to 1.18] .250
OR_adj_ [95% CI] * p*	1 (ref)	0.46 [0.18 to 1.22] .119	1.39 [0.80 to 2.42] .241	0.62 [0.33 to 1.18] .145
Used cessation support				
%	56.1	54.0	54.2	52.5
OR [95% CI] * p*	1 (ref)	0.92 [0.56 to 1.51] .732	0.93 [0.64 to 1.34] .700	0.87 [0.65 to 1.18] .371
OR_adj_ [95% CI] * p*	1 (ref)	0.95 [0.50 to 1.81] .884	1.10 [0.68 to 1.78] .710	0.98 [0.63 to 1.53] .923
Not currently smoking				
%	18.3	15.9	15.3	14.8
OR [95% CI] * p*	1 (ref)	0.85 [0.43 to 1.67] .638	0.82 [0.49 to 1.35] .433	0.76 [0.50 to 1.16] .204
OR_adj_ [95% CI] * P*	1 (ref)	0.60 [0.22 to 1.65] .323	0.88 [0.46 to 1.69] .695	0.80 [0.43 to 1.50] .490

CI, confidence interval; OR, odds ratio; OR_adj_, adjusted for age, ethnicity, social grade, marital status, disability, and survey year.

**Table 3.** Smoking and Cessation Behavior in Relation to Sexual Orientation in Women

	Heterosexual	Bisexual	Lesbian	Prefer-not-to-say
All adults (*n*)	52 457	686	1277	2910
Cigarette smoking				
%	16.9	29.8	18.3	14.5
OR [95% CI] * P*	1 (ref)	2.09 [1.77 to 2.47] <.001	1.10 [0.95 to 1.27] .189	0.84 [0.76 to 0.93] .001
OR_adj_ [95% CI] * P*	1 (ref)	1.64 [1.33 to 2.03] <.001	0.98 [0.83 to 1.17] .837	0.85 [0.72 to 0.99] .041
E-cigarette use				
%	4.9	8.8	4.5	4.0
OR [95% CI] * P*	1 (ref)	1.85 [1.41 to 2.42] <.001	0.91 [0.70 to 1.19] .509	0.80 [0.66 to 0.97] .020
OR_adj_ [95% CI] * P*	1 (ref)	1.30 [0.92 to 1.84] .141	0.73 [0.53 to 1.01] .057	0.80 [0.61 to 1.06] .126
Current smokers (*n*)	8817	204	232	419
Cigarettes per day				
Mean (SD)	10.6 (7.3)	8.7 (7.8)	11.1 (7.6)	10.6 (8.0)
*B* [95% CI] * p*	Ref	−2.08 [−3.13 to −1.03] <.001	0.40 [−0.57 to 1.38] .418	−0.01 [−0.74 to 0.72] .984
*B*_adj_ [95% CI] * p*	Ref	−0.88 [−2.07 to 0.31] .148	0.58 [−0.47 to 1.63] .279	−0.33 [−1.33 to 0.68] .525
First smoke within 30 min of waking				
%	48.1	33.8	44.8	47.6
OR [95% CI] * p*	1 (ref)	0.55 [0.41 to 0.74] <.001	0.87 [0.67 to 1.13] .300	0.98 [0.81 to 1.19] .841
OR_adj_ [95% CI] * p*	1 (ref)	0.66 [0.45 to 0.95] .026	0.97 [0.71 to 1.33] .862	0.87 [0.65 to 1.18] .376
High motivation to stop				
%	16.4	15.7	18.6	13.8
OR [95% CI] * p*	1 (ref)	0.96 [0.66 to 1.40] .824	1.17 [0.84 to 1.64] .356	0.82 [0.62 to 1.09] .170
OR_adj_ [95% CI] * p*	1 (ref)	0.91 [0.56 to 1.46] .688	1.24 [0.85 to 1.83] .267	0.94 [0.63 to 1.41] .767
E-cigarette use				
%	20.2	22.5	15.9	18.1
OR [95% CI] * p*	1 (ref)	1.13 [0.81 to 1.58] .460	0.75 [0.52 to 1.07] .107	0.88 [0.68 to 1.13] .301
OR_adj_ [95% CI] * p*	1 (ref)	0.94 [0.60 to 1.45] .767	0.66 [0.42 to 1.02] .063	0.94 [0.64 to 1.36] .725
Former smokers (*n*)	8880	88	186	459
E-cigarette use				
%	8.0	11.4	9.7	7.0
OR [95% CI] * p*	1 (ref)	1.49 [0.77 to 2.89] .233	1.23 [0.75 to 2.01] .419	0.85 [0.59 to 1.23] .385
OR_adj_ [95% CI] * p*	1 (ref)	0.70 [0.31 to 1.61] .403	0.99 [0.56 to 1.73] .966	0.84 [0.50 to 1.43] .520
Never-smokers (*n*)	34 719	393	853	2018
E-cigarette use				
%	0.3	1.0	0.4	0.4
OR [95% CI] * p*	1 (ref)	4.30 [1.61 to 11.47] .004	1.37 [0.43 to 4.38] .596	1.51 [0.72 to 3.16] .274
OR_adj_ [95% CI] * p*	1 (ref)	4.03 [1.46 to 11.09] .007	0.92 [0.24 to 3.55] .899	1.05 [0.35 to 3.17] .933
Past-year smokers (*n*)	9243	210	246	422
Past-year quit attempt				
%	35.1	38.1	36.2	28.9
OR [95% CI] * p*	1 (ref)	1.14 [0.86 to 1.51] .360	1.04 [0.80 to 1.36] .749	0.75 [0.61 to 0.93] .009
OR_adj_ [95% CI] * p*	1 (ref)	1.16 [0.82 to 1.64] .396	1.09 [0.80 to 1.49] .577	0.82 [0.59 to 1.13] .230
Past-year smokers who made a quit attempt (*n*)	3242	81	89	122
Motivated by GP advice				
%	17.5	22.5	19.6	23.0
OR [95% CI] * p*	1 (ref)	1.35 [0.79 to 2.30] .273	0.93 [0.53 to 1.64] .795	1.42 [0.93 to 2.19] .108
OR_adj_ [95% CI] * p*	1 (ref)	2.06 [1.09 to 3.89] .026	1.17 [0.61 to 2.22] .641	1.43 [0.74 to 2.75] .286
Motivated by health concerns				
%	45.3	45.0	48.3	45.9
OR [95% CI] * p*	1 (ref)	1.00 [0.64 to 1.56] .986	1.13 [0.74 to 1.73] .563	1.01 [0.70 to 1.45] .955
OR_adj_ [95% CI] * p*	1 (ref)	0.95 [0.54 to 1.65] .844	0.99 [0.60 to 1.64] .978	1.07 [0.62 to 1.87] .808
Motivated by cost				
%	20.8	22.2	25.8	17.2
OR [95% CI] * p*	1 (ref)	1.07 [0.63 to 1.84] .795	1.30 [0.80 to 2.12] .285	0.80 [0.50 to 1.29] .364
OR_adj_ [95% CI] * p*	1 (ref)	1.02 [0.52 to 2.01] .956	1.42 [0.81 to 2.50] .226	0.78 [0.38 to 1.61] .498
Used cessation support				
%	59.4	43.8	53.9	58.2
OR [95% CI] * p*	1 (ref)	0.53 [0.34 to 0.83] .006	0.79 [0.52 to 1.21] .275	0.94 [0.65 to 1.36] .745
OR_adj_ [95% CI] * p*	1 (ref)	0.52 [0.29 to 0.92] .024	0.80 [0.49 to 1.33] .398	0.89 [0.51 to 1.56] .683
Not currently smoking				
%	17.2	15.0	13.5	13.8
OR [95% CI] * p*	1 (ref)	0.87 [0.47 to 1.60] .646	0.75 [0.40 to 1.38] .355	0.75 [0.44 to 1.27] .288
OR_adj_ [95% CI] * p*	1 (ref)	0.74 [0.33 to 1.67] .472	0.61 [0.28 to 1.31] .203	0.45 [0.17 to 1.15] .096

CI, confidence interval; OR, odds ratio; OR_adj_, adjusted for age, ethnicity, social grade, marital status, disability, and survey year.

#### Smoking Prevalence

**Figure 1.**
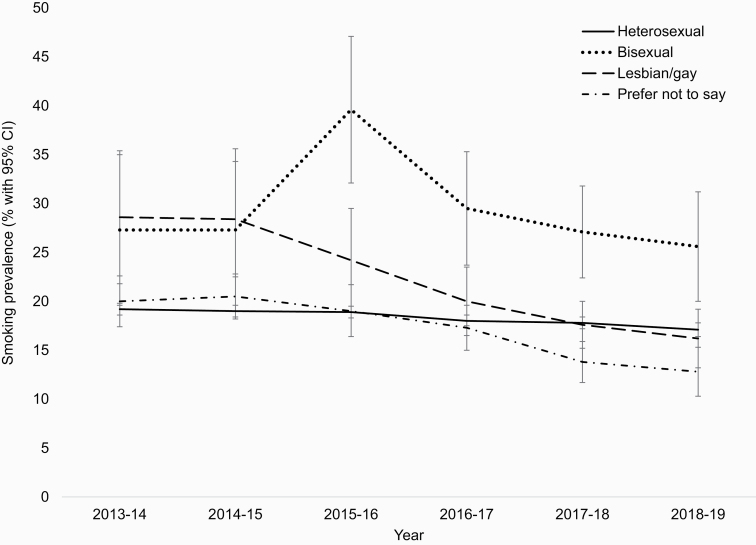
Annual trends in smoking prevalence among adults in England by sexual orientation. Years run from July through June, with the exception of 2018–2019 which runs from July through February.

Smoking prevalence was higher among those who identified as bisexual (28.2% in men, 29.8% in women) than those who identified as heterosexual (20.0% in men, 16.9% in women). These differences were significant even after adjustment for covariates (men: OR_adj_ = 1.41, 95% CI = 1.11 to 1.79; women: OR_adj_ = 1.64, 95% CI = 1.33 to 2.03). No significant differences in smoking prevalence were observed between gay/lesbian and heterosexual men or women on aggregated data, with adjusted models providing moderate evidence for the null hypothesis (BF = 0.3 for both men and women). However, the difference between these groups appeared to change over the study period (see Figure 1, described in more detail). In women, those who preferred not to disclose their sexual orientation had lower odds of smoking, which remained after adjustment (OR_adj_ = 0.85, 95% CI = 0.72 to 0.99). There was no significant difference between men who preferred not to say and heterosexual men, with data proving insensitive (BF = 0.5).

Figure 1 shows annual trends in smoking prevalence between 2013/2014 and 2018/2019 for those identifying as gay/lesbian, bisexual, heterosexual, and those who preferred not to disclose their sexual orientation. Among heterosexuals, there was a steady decline in smoking prevalence over the study period, from 19.2% in 2013/2014 to 17.1% in 2018/2019. Smoking prevalence among those who identified as gay/lesbian or bisexual was notably higher in 2013/2014, at 28.6% and 27.3%, respectively. Over the study period, there appeared to be a more rapid decline in prevalence among gay/lesbian participants than was seen in heterosexuals, reaching a more comparable 16.2% in 2018/2019, but smoking prevalence was relatively stable in those who identified as bisexual, remaining elevated at 25.6% in 2018/2019. Among those who preferred not to disclose their sexual orientation, prevalence of smoking in 2013/2014 was similar to that of heterosexuals (20.0%), but the trend over time was similar to that of the gay/lesbian group, such that prevalence in 2018/2019 was the lowest of the four groups (12.8%).

#### Use of E-Cigarettes

Among the entire adult population, e-cigarette use was more prevalent among bisexual men (8.8%) and bisexual women (8.8%) than heterosexuals (5.9% in men, 4.9% in women), but differences were nonsignificant after adjustment for covariates. Among current smokers, e-cigarette use was not significantly associated with sexual orientation in men or women. Among former smokers, e-cigarette use did not differ significantly by sexual orientation in women but was more prevalent in gay than heterosexual men even after adjustment for covariates (OR_adj_ = 2.05, 95% CI = 1.29 to 3.25). E-cigarette use among never-smokers was rare, reported by ≤1% of each sexual orientation group in both men and women. There were no significant differences in e-cigarette use among never-smokers by sexual orientation in men, but in women, bisexual never-smokers (4/393) had significantly higher odds of reporting e-cigarette use than heterosexual never-smokers (88/34 719), which persisted after adjustment for covariates (OR_adj_ = 4.03, 95% CI = 1.46 to 11.09).

#### Level of Cigarette Addiction

Among current smokers, mean daily cigarette consumption was significantly lower in gay (10.5 cigarettes/day, *B*_adj_ = −1.26, 95% CI = −2.46 to −0.06) and bisexual men (10.7 cigarettes/day, *B*_adj_ = −2.41, 95% CI = −4.06 to −0.75) than heterosexual men (11.8 cigarettes/day). There was a similar difference between bisexual and heterosexual women (8.7 vs. 10.6 cigarettes/day), but this was not statistically significant after adjustment for covariates. No differences in daily cigarette consumption were observed between prefer-not-to-say men and heterosexual men, or between lesbian or prefer-not-to-say women and heterosexual women.

The proportion of current smokers who reported smoking their first cigarette of the day within 30 min of waking did not differ significantly by sexual orientation in men. However, in women, there was lower prevalence in those who identified as bisexual than in heterosexuals (33.8% vs. 48.1%). This difference was statistically significant after adjustment for covariates (OR_adj_ = 0.66, 95% CI = 0.45 to 0.95).

#### Motivation to Stop Smoking, Quit Qttempts, Motives, and Success Rate

Among current smokers, there was no significant difference in motivation to stop by sexual orientation in either men or women, with the data proving insensitive (BF for comparisons between heterosexuals and those identifying as bisexual, lesbian/gay, and prefer-not-to-say = 1.2, 0.5, and 0.6, respectively, for men, and 0.7, 1.1, and 0.6, respectively, for women).

Among past-year smokers, the proportion who had made a serious attempt to quit in the past year did not differ significantly after adjustment for covariates with the data proving insensitive (BF for comparisons between heterosexuals and those identifying as bisexual, gay/lesbian, and prefer-not-to-say = 0.9, 1.3, and 1.8, respectively, for men and 0.8, 0.6, and 1.1, respectively, for women).

Among smokers who had made a quit attempt in the past year, men who identified as gay, bisexual, or preferred not to disclose their sexual orientation were more likely than heterosexual men to cite advice from a GP as a motive for their most recent quit attempt (OR_adj_ range = 2.08–5.21), but they were less likely to cite health concerns as a motive (OR_adj_ range = 0.51–0.64). Bisexual women were also more likely than heterosexual women to cite advice from a GP as a motive for their most recent quit attempt (OR_adj_ = 2.06, 95% CI = 1.09 to 3.89), but other groups did not differ significantly. There were no significant differences by sexual orientation in the proportion of women citing health concerns as a motive for quitting, or the proportion of men or women citing cost as a motive for quitting.

Use of cessation support in the most recent quit attempt did not differ significantly by sexual orientation in men, but was less commonly reported by bisexual women than heterosexual women (OR_adj_ = 0.52, 95% CI = 0.29 to 0.92). The success rate of quit attempts did not differ significantly by sexual orientation in either men or women.

## Discussion

In a representative sample of more than 100 000 men and women in England, smoking rates were higher among those who identified as bisexual than those who identified as heterosexual, after adjustment for a range of sociodemographic covariates, but were not significantly higher for those who identified as lesbian/gay and were significantly lower in women but not men who preferred not to disclose their sexual orientation. Among smokers, gay and bisexual men and bisexual women appeared to be less addicted to cigarettes. Prevalence of e-cigarette use was similar across sexual orientations for all adults and current smokers but was significantly higher in bisexual than heterosexual female never-smokers and in gay than heterosexual male former smokers. Motivation to stop smoking, past-year quit attempts, and the success rate of quit attempts did not differ significantly by sexual orientation in either men or women, although there were some differences in motives for quitting.

A number of previous studies have documented higher rates of smoking among LGB individuals,^5–15^ which has led to these sexual minority groups being highlighted in NICE guidance as a priority for smoking cessation efforts. However, a previous analysis of STS data indicated that the higher prevalence of smoking among LGB groups could be explained by differences between these groups and heterosexuals in other sociodemographic factors, such as age, ethnic background, and socioeconomic position.^16^ With data now available for a much larger sample of STS participants, we found differences in prevalence between bisexual and heterosexual participants remained statistically significant in men and women, even after adjustment for these variables (although other potentially relevant factors, such as education, Internet use, and region were not included). However, contrary to other studies but in agreement with the previous STS analysis, we found moderate evidence for there being no difference in smoking prevalence between gay/lesbian people and heterosexual people.

A key finding was that the disparity in smoking prevalence between gay/lesbian people and heterosexuals appears to be decreasing over time. In 2013/2014, smoking prevalence was 49% higher in gay/lesbian than heterosexual men and women (28.6% vs. 19.2%, respectively); in 2018/2019, it was slightly lower (16.2% vs. 17.1%). This narrowing of differences could reflect societal changes making the environment more similar across sexual orientations. Marriage equality came into power in 2014 in England and Wales, changing the social landscape for LGB individuals. Government bodies have emphasized the need to tackle health inequalities in sexual minority groups. For example, in 2014, Public Health England published an action plan to promote health and wellbeing in gay and bisexual men, which listed closing the gap in smoking as a key priority.^33^ In addition, it is possible that the ban on smoking in public places that was implemented in England in 2007 had a greater impact on smoking in sexual minority groups, who traditionally use bars and other social recreational spaces where smoking used to be commonplace as safe places of gathering.^18^ To our knowledge, there has been very little research into trajectories of sexual orientation-related smoking disparities, making it difficult to establish whether the same pattern has occurred in other countries with similarly progressive attitudes towards sexual minority groups. However, biennial surveys of lesbian, bisexual, and queer women in Australia documented a marked drop in smoking prevalence in these groups between 2016 and 2018 (from 30% to 22%) following a period of much slower decline since 2004.^34,35^ In addition, a recent study from the United States reported some evidence of a narrowing of disparities in cigarette use among adolescents; results indicated that while disparities had remained broadly stable from 2005 to 2015, disparities in heavy use and lifetime use had reduced for bisexual boys and lesbian girls.^9^

While one may expect there to be a similar decline across all sexual minority groups if this was being driven by positive changes in the social landscape, our results showed very different patterns of smoking prevalence between gay/lesbian and bisexual people. The gap has narrowed between gay/lesbian and heterosexual people but bisexual people in England remain significantly more likely to smoke. This is consistent with previous research that has disaggregated gay and bisexual men and lesbian and bisexual women and found that bisexual people are more likely to engage in health risk behaviors (eg, alcohol, tobacco, and other drug use, unprotected sex) and suffer poor mental and physical health.^20,36–38^ It has been suggested that these differences may be the result of additional stressors placed on bisexual people as a result of “double discrimination” from both heterosexual and gay/lesbian people.^36^ These results emphasize the need to consider sexual minority groups individually as opposed to one homogenous group; rather than all sexual minorities necessarily being a high prevalence group needing special attention, there may need to be greater focus on bisexual men and women.

In addition to smoking prevalence, we examined differences in a range of other smoking-related characteristics, including level of addiction, motivation to stop, and aspects relating to quit attempts. These have been largely unexplored in the existing literature, with only the previous STS study^16^ reporting on differences in these variables in relation to sexual orientation. In this previous study, unadjusted models indicated that there were no significant differences in cigarette consumption, motivation to stop smoking, or quit attempts by sexual orientation in either men or women, although bisexual women had lower levels of addiction.^16^ Whether differences persisted after adjustment for covariates was not explored. The present study elaborated on these results, providing a more detailed examination of smoking and quitting behavior, including novel data on trends in prevalence over time, use of e-cigarettes, motives for quitting, and the success rate among quitters. Importantly, we also adjusted for relevant covariates to provide insight into differences associated with sexual orientation over and above other related sociodemographic characteristics. In line with the previous study, we found no significant differences in motivation to stop smoking or quit attempts among smokers. We confirmed the finding that bisexual women were less addicted than heterosexual women, indicated by lower odds of smoking within the first 30 min of waking. We also showed that gay and bisexual men smoked fewer cigarettes per day, on average, than heterosexual men. This indicates that men from sexual minority groups may also have lower levels of addiction, although lower cigarette consumption can be driven by cost as well as dependence.

A previous study reported higher prevalence of e-cigarette use among LGB men and women.^6^ We also identified significant independent associations between sexual orientation and use of e-cigarettes, but findings differed by gender and smoking status. Among male former smokers, current e-cigarette use was reported by a substantial minority (9%) with gay men twice as likely to report using e-cigarettes than heterosexual men. It is possible that adoption of newer technology is greater in this group. The higher prevalence of e-cigarette use may have contributed to the more rapid decline in smoking prevalence observed in this group. Among female never-smokers, current use was rare (<1%) but bisexual women were four times as likely to report using e-cigarettes than heterosexual women. However, use of e-cigarettes did not differ significantly by sexual orientation in current smokers of either sex.

There was also some evidence in the present results of differences in motives for quitting among smokers who reported having made a serious quit attempt in the past 12 months. Sexual minority groups (men who identified as gay, bisexual, or preferred not to say, and women who identified as bisexual) were more likely to report GP advice as a motivating factor, which could have been driven by potentially more visits to the GP among sexual minority groups,^39^ although men from sexual minority groups were less likely to report being motivated to quit by health concerns. The proportion citing cost as a motive for quitting did not differ by sexual orientation in either men or women. Given the small number of smokers who reported a quit attempt in LGB and prefer-not-to-say groups, confidence intervals for these results were wide, so some degree of caution should be exercised in interpreting these findings. However, if systemic differences in motives for quitting do exist between groups with differing sexual orientations, this could be important for informing the development of targeted interventions to promote cessation among sexual minorities. Further research, perhaps of a qualitative nature, is needed to explore this issue in more detail.

This study had strengths, including a large sample representative of the adult population in England,^24^ and adjustment for a range of relevant covariates. However, it was not without limitations. While the Smoking Toolkit Study has been demonstrated to represent the entire adult population in England on sociodemographic and smoking characteristics,^23^ the extent to which it is representative of sexual minorities has not been established. This is a challenging methodological issue that affects all surveys given information on sexual identity is not collected in the English census. Other nationally representative surveys provide broadly similar estimates of the prevalence of sexual minorities,^40^ although comparison with the most recent National Surveys of Sexual Attitudes and Lifestyles (Natsal) in Britain showed similar rates of bisexuality but lower prevalence of gay men (1.5% vs. 2.5%) and lesbian women (1.0% vs. 2.2%) than we observed in our sample.^41^ Despite the large sample, the number of men and women identifying as LGB was relatively small, limiting statistical power to detect subtle differences. Indeed, Bayes factors indicated that data were insensitive to distinguish between the null and alternative hypothesis for some of our outcomes of interest. Items relating to the most recent quit attempt (motives, use of cessation support, past-year quit attempts) relied on recall of events up to 12 months prior, introducing scope for bias. A substantial proportion of participants opted not to disclose their sexual orientation, which may have led to under- or over-estimation of differences between heterosexual and LGB groups. No data are collected in STS on gender identity, so we were unable to explore smoking behavior in trans-identifying people, who also fall under the group of sexual minorities identified by NICE as high-risk for smoking.^3^ The existing literature on smoking in transgender versus cisgender groups is mixed and requires further exploration.^7,8,42^

## Conclusions

In conclusion, the disparity in smoking prevalence between adults in England who identify as lesbian, gay, or bisexual and those who identify as heterosexual has decreased over recent years. Some aspects of smoking and quitting behavior still differ significantly by sexual orientation—notably higher smoking prevalence among those who identify as bisexual and lower smoking prevalence among women who prefer not to say, but lower levels of addiction and different motives for quitting among some LGB groups, compared with those who identify as heterosexual—however, we found no evidence of differences in motivation to stop smoking, quit attempts, or quit success after controlling for other relevant sociodemographic characteristics.

## Supplementary Material

A Contributorship Form detailing each author’s specific involvement with this content, as well as any supplementary data, are available online at https://academic.oup.com/ntr.

ntaa042_suppl_Supplementary_Taxonomy_Form
